# Development and Bioactivity of Zinc Sulfate Cross-Linked Polysaccharide Delivery System of Dexamethasone Phosphate

**DOI:** 10.3390/pharmaceutics15102396

**Published:** 2023-09-28

**Authors:** Natallia V. Dubashynskaya, Anton N. Bokatyi, Andrey S. Trulioff, Artem A. Rubinstein, Igor V. Kudryavtsev, Yury A. Skorik

**Affiliations:** 1 Institute of Macromolecular Compounds of the Russian Academy of Sciences, Bolshoi VO 31, 199004 Saint Petersburg, Russia; qwezakura@yandex.ru (A.N.B.); yury_skorik@mail.ru (Y.A.S.); 2 Institute of Experimental Medicine, Acad. Pavlov St. 12, 197376 Saint Petersburg, Russia; trulioff@gmail.com (A.S.T.); arrubin6@mail.ru (A.A.R.); igorek1981@yandex.ru (I.V.K.)

**Keywords:** dexamethasone phosphate, zinc sulfate, polysaccharides, chitosan, hyaluronan, ocular delivery systems, mucoadhesion, anti-inflammatory activity

## Abstract

Improving the biopharmaceutical properties of glucocorticoids (increasing local bioavailability and reducing systemic toxicity) is an important challenge. The aim of this study was to develop a dexamethasone phosphate (DexP) delivery system based on hyaluronic acid (HA) and a water-soluble cationic chitosan derivative, diethylaminoethyl chitosan (DEAECS). The DexP delivery system was a polyelectrolyte complex (PEC) resulting from interpolymer interactions between the HA polyanion and the DEAECS polycation with simultaneous incorporation of zinc ions as a cross-linking agent into the complex. The developed PECs had a hydrodynamic diameter of 244 nm and a ζ-potential of +24.4 mV; the encapsulation efficiency and DexP content were 75.6% and 45.4 μg/mg, respectively. The designed DexP delivery systems were characterized by both excellent mucoadhesion and prolonged drug release (approximately 70% of DexP was released within 10 h). In vitro experiments showed that encapsulation of DexP in polysaccharide nanocarriers did not reduce its anti-inflammatory activity compared to free DexP.

## 1. Introduction

Various ocular pathologies of inflammatory genesis have a negative impact on the quality of life of patients and can lead to blindness [1]. Topical application of drugs is the preferred way to treat ocular diseases due to its non-invasiveness and safety [2]. Disadvantages of traditional ocular anti-inflammatory dosage forms (eye drops and ointments) are related to the rapid release of active pharmaceutical ingredients (APIs) and their subsequent rapid elimination from the site of administration due to the unique features of the anatomy and physiology of the eye (the tear film barrier, nasolacrimal duct drainage, constant rapid tear flow, and rapid precorneal clearance) [3,4,5,6], resulting in reduced bioavailability [7,8]. In addition, the anatomical corneal barrier and the low permeability of the cornea and sclera make topical application less effective for the treatment of posterior segment diseases [9]. The problem of rapid drug release from the dosage form can be solved by using polymeric solvents (e.g., cellulose derivatives, hyaluronic acid, etc.), which impart the necessary viscosity to the solution and thus modify the drug release [10,11]. However, these systems are not suitable for programmed and controlled drug release and corneal permeability improvement; in addition, they are not convenient to use because of the need for frequent application, resulting in low patient compliance [12].

One strategy to improve the biopharmaceutical properties of known drugs is the development of nanotechnology-based drug delivery systems, such as polymeric nano- and microparticles [1,10,13,14]. The particles in the form of interpolymer polyelectrolyte complexes (PECs) based on biopolymers (e.g., hyaluronic acid (HA), chitosan and its derivatives, etc.) are an attractive choice for the ocular delivery of anti-inflammatory drugs [15]. The incorporation of drug molecules into PECs of different structures allows the following: (i) to ensure selective targeting of the damaged tissues, (ii) to improve local bioavailability, (iii) to increase the residence time of the dosage form at the target site due to the mucoadhesive properties of biopolymers, (iv) to control the release rate from the polymer matrix, and (v) to reduce the degree and frequency of side effects [15,16,17]. In addition, the procedure for obtaining PECs is simple, convenient, and inexpensive and does not require the use of toxic reagents [1,18].

The physicochemical properties of polymeric particles, such as size, charge, and surface modification by targeting ligands (e.g., anti-VEGF antibodies [19], targeting peptide ICAM-1 [20], mannose [21], etc.), affect the efficiency of drug delivery and the efficacy of ocular disease treatment [22]. In this case, particles 50–400 nm in diameter are the preferred size for ophthalmic drug delivery because they provide more effective mucoadhesion and rapid penetration through the ocular barriers to the target site with less ocular irritation [10,23,24]. In addition, cationic nanocarriers have a longer residence time on the ocular mucosa due to their interaction with negatively charged mucus components, resulting in an enhanced ability to penetrate the drug into the eye [25,26]. On the other hand, the positive charge of the surface may prevent the particles from penetrating through the sclera and diffusing into the vitreous body due to their electrostatic binding with negatively charged components of these tissues [27]. By varying the conditions of PEC formation, it is possible to obtain polymeric carriers with desired physicochemical (size and surface charge) and pharmaceutical (rate and pattern of drug release) properties to improve ocular drug delivery, including anti-inflammatory agents of glucocorticoid nature, i.e., dexamethasone [28,29].

The disadvantages of biopolymer-based PECs are the relatively fast release of the drug (on average within 1–3 h) due to the disruption of ionic interactions between macromolecules under physiological conditions by the effect of pH and ionic strength [30]. This problem can be overcome by using different cross-linking agents such as metal ions (Zn^2+^, Ca^2+^, etc.) [31,32]. For instance, Tiyaboonchai et al. [33] used zinc sulfate to crosslink amphotericin B-containing nanoparticles based on polyethyleneimine and dextran. The introduction of zinc sulfate (25–50 mM) into the polyethyleneimine/dextran system in a 2:1 ratio resulted in a reduction in particle size from 800 nm to 300 nm and an increase in drug encapsulation efficiency from 70% to 80–90%. The Zn^2+^ ions can thus act as a reinforcing agent by cross-linking the polymer components. In addition, zinc-reinforced particles showed a drug release delay of up to 40% within 1 h.

Our previous studies have shown that polyanionic HA and polycationic diethylaminoethyl chitosan (DEAECS) with high degrees of substitution are promising for the formation of PECs [29]. In this case, stable complexes are formed whose size and charge depend on both the ratio of polymers and the order of mixing. The most stable PECs were obtained by mixing DEAEC and HA in ratios of 1:5 and 2:5. The hydrodynamic diameter of the obtained particles was 120–300 nm, and their surface charge ranged from −10 mV to −23 mV. In addition, according to our previous studies [30], the introduction of 20% DEAECS from the HA mass prolonged the release of colistin compared to the DEAECS-free complex by increasing the colloidal stability of the particles. In addition, both DEAECS and HA have attractive biomedical properties and are biodegradable, biocompatible, and non-toxic water-soluble polymers [34,35,36,37].

HA is a targeting ligand due to its high affinity for the CD44 and stabiliin-2 receptors, which are overexpressed at sites of inflammation and on the surface of immunocompetent cells (T and B lymphocytes and macrophages) [38,39,40], and chitosan and its cationic derivatives increase the permeability of drug molecules across the corneal surface due to their mucoadhesive properties and ability to open tight junctions [41]. For example, Mohamed et al. [42] developed chitosan nanoparticles loaded with the nonsteroidal anti-inflammatory drug meloxicam by electrostatic interaction between cationic chitosan and anionic drug using 0.25% sodium tripolyphosphate solution as a cross-linking agent. The resulting particles had a size of 200 to 600 nm, a ζ-potential of 25–54 mV, and an encapsulation efficiency of 70–90%. An in vitro study demonstrated sustained drug release within 72 h in PBS (pH 7.4). An ex vivo experiment demonstrated improved permeability of encapsulated meloxicam through both the cornea and sclera of rabbits compared to free drug. In in vivo studies, the dispersion of the obtained PECs showed enhanced anti-inflammatory activity and no ocular irritation compared to the solution of meloxicam eye drops. In another study [43], Ricci et al. developed mucoadhesive polyelectrolyte particles for ocular delivery of the nonsteroidal anti-inflammatory drug indomethacin based on chitosan and sulfobutyl ether-β-cyclodextrin with a diameter of 350 nm and a ζ-potential of +18 mV. The resulting particles were additionally coated with thiolated low-molecular-weight HA to reverse the surface charge to negative. The positively charged chitosan particles had excellent corneal permeability, making them attractive nanoplatforms for indomethacin delivery to the posterior segment of the eye. On the other hand, thiolated hyaluronic acid-coated particles showed prolonged residence time in the conjunctival sac, making them an optimal drug delivery system for the treatment of inflammatory diseases of the anterior segment of the eye.

The aim of the present work was to develop a suitable system to improve the local ocular delivery of glucocorticoids based on HA-DEAECS PECs with prolonged release and anti-inflammatory activity. Water-soluble dexamethasone phosphate (DexP) was chosen as a model glucocorticoid. DexP is one of the most effective drugs in the treatment of inflammatory diseases, but its high systemic toxicity, the need for long-term administration, and dose-dependent severe side effects limit its medical use [44]. The encapsulation of DexP in mucoadhesive polysaccharide-based PECs ensures its controlled release and targeted delivery and increases the residence time on the ocular mucosa, thereby reducing the dosage and frequency of side effects [45,46]. Furthermore, the use of zinc sulfate as a cross-linking agent can be beneficial not only for prolonging DexP release but also for potentiating/synergizing its pharmacological action through its own biological activity (including anti-inflammatory, antimicrobial, and wound healing) [28,47].

## 2. Materials and Methods

### 2.1. Materials and Reagents

Sodium hyaluronate with a viscosity average MW of 180,000 [48] was used in this work. DEAECS, with the values of degree of substitution 83% and degree of quaternization 14%, was previously synthesized and characterized [29]. The starting material for the synthesis of DEAECS was crab shell chitosan with an average MW of 37,000 and a degree of deacetylation (DDA) of 74% [49].

DexP, phosphate-buffered saline (PBS), zinc sulfate, mucin (type II), periodic acid, basic fuchsin, and sodium pyrosulfite were from Sigma-Aldrich (St. Louis, MI, USA), and the 1 M hydrochloric acid solution was from Acros Organics (Waltham, MA, USA).

### 2.2. General Methods

The hydrodynamic diameter (Dh) and the ζ-potential were determined by dynamic and electrophoretic light scattering (DLS and ELS), respectively, using a Compact-Z instrument (Photocor, Moscow, Russia) with a 659.7 nm He-Ne laser at 25 mV power and a detection angle of 90°. The polydispersity index (PDI) was determined by cumulants’ analysis of the autocorrelation function using DynaLS software v. 2 (SoftScientific, Tirat Carmel, Israel, http://www.softscientific.com/science/downloads.html#evals (accessed on 3 September 2023)).

UV–VIS spectra were obtained with a UV-1700 PharmaSpec spectrophotometer (Shimadzu, Kyoto, Japan).

Quantification of zinc and phosphorus was performed by inductively coupled plasma atomic emission spectroscopy (ICP-AES) using a Shimadzu Icpe-9820 spectrometer (Shimadzu, Kyoto, Japan).

Particle morphology was examined by scanning electron microscopy (SEM) using a Tescan Mira 3 scanning electron microscope (Tescan, Brno, Czech Republic). The samples were placed on double-sided carbon tape and dried in a vacuum oven for 24 h. Images were acquired in the secondary electron mode at an accelerating voltage of 20 kV and an operating current of 543.3 pA. The distance between the sample and the detector was approximately 6 mm.

### 2.3. Preparation of PECs

**Scheme 1:**
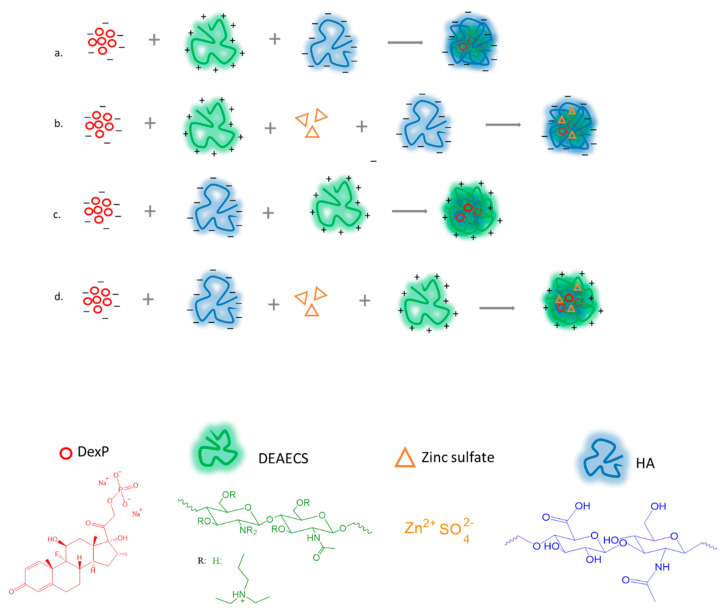
Preparation of PECs: DexP-DEAECS-HA (**a**), DexP-DEAECS-Zn-HA (**b**), DexP-HA-DEAECS (**c**), and DexP-HA-Zn-DEAECS (**d**).

Solutions of HA (10 mg/mL), DEAECS (10 mg/mL), ZnSO_4_ (1 mg/mL), and DexP (1 mg/mL) were prepared in bi-distilled water. PECs were obtained by mixing the components according to the following procedures (Scheme 1):

(i) To the DexP solution, DEAECS solution was added, followed by the addition of HA solution (DexP-DEAECS-HA; Scheme 1a);
(ii) To the DexP solution, DEAECS solution was added, then zinc sulfate solution was added, followed by the addition of HA solution (DexP-DEAECS-Zn-HA; Scheme 1b);
(iii) To the DexP solution, HA solution was added, followed by the addition of DEAECS solution (DexP-HA-DEAECS; Scheme 1c);
(iv) To the DexP solution, HA solution was added, then zinc sulfate solution was added, followed by the addition of DEAECS solution (DexP-HA-Zn-DEAECS; Scheme 1d).

All solutions were added dropwise with a 23G needle under ultrasound treatment conditions (at 20 W, pulse-on 3 s and pulse-off 7 s, total 180 s) using a Bandelin Sonopuls mini 20 probe ultrasonicator (Bandelin Electronics, Berlin, Germany). The resulting systems were concentrated by ultrafiltration at 4500 rpm using a Vivaspin^®^ Turbo 4 centrifugal concentrator with a pore size of 10,000 MWCO (Sartorius AG, Göttingen, Germany) to separate the non-encapsulated components (DexP and ZnSO_4_). Various PEC formation parameters are shown in Table 1. The formed PECs were freeze-dried using a 10 N freeze dryer (Fanbolun Ltd., Guangzhou, China).

### 2.4. Encapsulation Efficiencies and DexP Content

Encapsulation efficiency (EE) and DexP content (μg/mg) were determined by measuring the concentration of unloaded DexP (indirect method). The PEC suspension was concentrated by ultrafiltration (see Section 2.3). The amount of encapsulated DexP in the PEC was calculated from the difference between the total amount of DexP used to prepare the PECs and the amount of DexP in the filtrate. The concentration of DexP in the filtrate was determined spectrophotometrically at a wavelength of 242 nm using a calibration curve (10 mm quartz cuvette, UV-visible spectrophotometer Shimadzu UV-1700 Pharma Spec, Japan). The results were calculated according to the following equations:

(1) $$\mathrm{EE}\;(\%)=\frac{\left(DexP\;mass\;total-DexP\;mass\;in\;the\;filtrate\right)\times 100}{DexP\;mass\;total}$$

(2) $$\mathrm{DexP}\;\mathrm{content}\;(\mu g/\mathrm{mg})=\frac{\left(Dex\;mass\;total-Dex\;mass\;in\;the\;filtrate\right)\times 1000}{PEC\;mass}$$

### 2.5. In Vitro DexP Release

The release test conditions were selected based on the FDA recommendation for dissolution methods for topical ophthalmic dosage forms [13]. A 10 mg sample was dispersed in PBS (2 mL, pH 7.4) and incubated at 32 °C. At specified time intervals, the nanosuspension was ultracentrifuged at 4500 rpm using a 10,000 MWCO Vivaspin^®^Turbo4 centrifugal concentrator, and the volume of dissolution medium was replenished with fresh PBS. The amount of DexP released was determined spectrophotometrically.

### 2.6. Mucoadhesion

The mucin binding efficiency was evaluated by mucin adsorption using the two-step periodic acid/Schiff colorimetric method [50,51]. Periodic acid was prepared as follows: 10 μL of 50% periodic acid was added to 7 mL of 7% acetic acid. Schiff’s reagent was prepared as follows: 100 mL of 1% aqueous basic fuchsin was added to 20 mL of 1 M HCl; the resulting mixture was decolorized twice for 5 min with 300 mg activated charcoal. Sodium pyrosulfite (0.1 g per 6 mL of Schiff’s reagent) was added just before use, and the resulting solution was incubated at 37 °C until it became colorless or pale yellow (about 90–100 min).

The calibration curve was constructed as follows: 200 μL of freshly prepared periodic acid was added to 2 mL of standard mucin solutions (0.02–0.08 mg/mL). The resulting solutions were incubated at 37 °C for 120 min to complete the periodate oxidation; then, colorless Schiff reagent (200 μL) was added and allowed to stand for 30 min at room temperature (the solution turned pink). The absorbance of the standards was measured at 565 nm.

Mucin solution (0.5 mg/mL; 1 mL) was added to the DexP-DEAECS-Zn-HA and DexP-HA-Zn-DEAECS (0.5 mg/mL; 10 mL) with magnetic stirring at 500 rpm, and the mixture was incubated at 37 °C for 60 min. The resulting mixture was centrifuged at 4500 rpm for 60 min, and the supernatant was used to measure the free mucin concentration using the calibration curve. A solution containing all the components of the analyzed solution, except for the analyte, was used as a reference solution. Mucoadhesiveness (mucin binding efficiency) was calculated using the following equation:

(3) $$\mathrm{Mucoadhesiveness}\;(\%)=\frac{\left(m_{o}-m_{s}\right)\times 100}{m_{o}}$$

where m_o_ is the initial mucin mass and m_s_ is the mucin mass in the supernatant.

### 2.7. Anti-Inflammatory Activity

Human monocytic leukemia cells (THP-1 cells) were used to study the in vitro effects of DexP and DexP-containing PECs. Cell line THP-1 was obtained from the Collection of Vertebrate Cell Cultures maintained by the Institute of Cytology of the Russian Academy of Sciences. THP-1 cells were cultured at 37 °C in a humidified atmosphere with 5% CO_2_ in RPMI-1640 medium (Biolot, St. Petersburg, Russia) supplemented with 10% (*v*/*v*) heat-inactivated fetal calf serum (FBS, Gibco Inc., Grand Island, NY, USA), 2 mM L-glutamine (Biolot, St. Petersburg, Russia), and 50 μg/mL gentamicin (Biolot, St. Petersburg, Russia), as previously described [13]. Primarily, we investigated the effects of DexP on cell viability, and flow cytometry based on YO-PRO-1/PI staining was performed to detect viable and apoptotic cells. YO-PRO-1 iodide (Molecular Probes, Eugene, OR, USA) was used at a final concentration of 250 nM, and propidium iodide (PI, Merck KGaA, Darmstadt, Germany) was used at a final concentration of 1 μM. Method principles and “gating strategy” were described previously [52]. A minimum of 10,000 THP-1 cells were analyzed per sample. Flow cytometry data were obtained using a Navios™ flow cytometer (Beckman Coulter, Beckman Coulter Inc., Indianapolis, IN, USA) equipped with 405, 488, and 638 nm lasers and analyzed using Navios software v.1.2 and Kaluza™ software v.2.0 (Beckman Coulter, Beckman Coulter Inc., Indianapolis, IN, USA). Data were presented as median and interquartile range, Me (Q25; Q75). Differences between groups were analyzed using a non-parametric Mann–Whitney U test with a value of *p* < 0.05.

Next, we investigated the ability of DexP, ZnSO_4_, and DexP/Zn^2+^-containing PECs to suppress in vitro activation of THP-1 cells. We activated THP-1 cells in vitro by adding recombinant human tumor necrosis factor-α protein (final concentration 2 ng/mL, BioLegend Inc., San Diego, CA, USA), while untreated THP-1 cells were used as a negative control. The test compounds (DexP, ZnSO_4_, DexP-HA-DEAECS, DexP-HA-Zn-DEAECS, DexP-DEAECS-HA, DexP-DEAECS-Zn-HA, and HA-DEAECS) were added to 200 μL THP-1 cell suspension (200 μL cell culture medium containing 1 × 105 cells in suspension) and incubated for 24 h in 96-well flat-bottom culture plates (Sarstedt, Germany). The concentrations of the compounds tested were equivalent to a DexP concentration of 0.1 μg/mL. The cells were then transferred to 75 mm × 12 mm flow cytometry tubes (Sarstedt, Germany) and washed with 4 mL sterile PBS (centrifugation at 300× *g* for 5 min). The resulting cell sediments were resuspended in 100 µL fresh sterile PBS and stained with mouse anti-human CD54-PE antibody (clone HA58, isotype—mouse IgG1, κ; BioLegend Inc., San Diego, CA, USA) for 15 min in the dark as described previously [14]. Finally, THP-1 cell samples were washed again and stained with DAPI (final concentration 1 μg/mL; BioLegend Inc., San Diego, CA, USA) to distinguish between live and dead cells. A minimum of 10,000 single THP-1 cells were collected per sample. Flow cytometry data were obtained using a Navios™ flow cytometer (Beckman Coulter Inc., CA, USA) equipped with 405, 488, and 638 nm lasers and analyzed using Navios software v.1.2 and Kaluza™ software v.2.0 (Beckman Coulter Inc., CA, USA). The intensity of CD54 expression was finally measured as mean fluorescence intensity (MFI) on the cell surface of viable THP-1 cells. Data were presented as median and interquartile range, Me (Q25; Q75). Differences between groups were analyzed using a non-parametric Mann–Whitney U test with a value of *p* < 0.05.

## 3. Results and Discussion

### 3.1. Preparation and Characterization of the PECs

**Figure 1:**
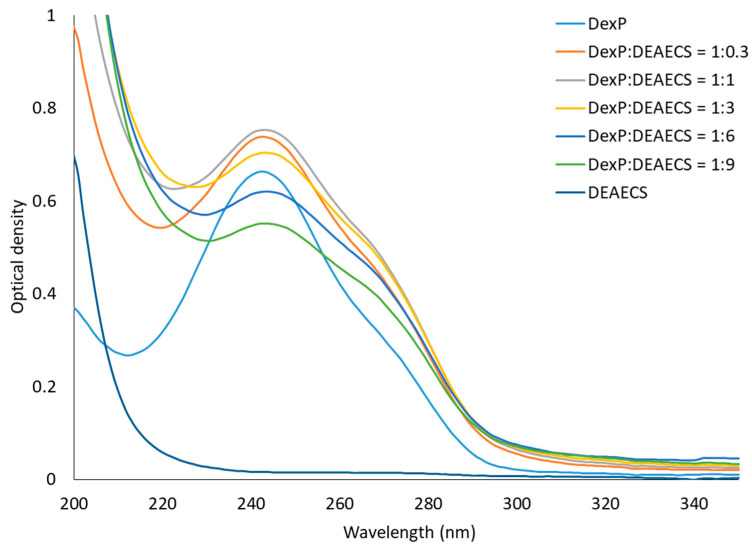
UV–VIS absorption spectra of mixtures of DEAECS and DexP at different ratios in water.

DEAECS is an alkylated derivative of chitosan with a high positive charge density. Typically, the substitution reaction proceeds through both amine and hydroxyl groups, and 0–15% of the diethylaminoethyl groups are alkylated to form quaternary ammonium groups [29]. The first step of our study was to investigate the interaction of the water-soluble cationic polymer DEAECS with negatively charged DexP molecules using spectrophotometry [53]. Titration of the DexP solution (0.025 mg/mL) with the DEAECS solution (0.3 mg/mL) showed a change in the shape of the DexP absorption spectrum and a decrease in absorption intensity with increasing polymer content (hypochromic effect), indicating that these components interact with each other (Figure 1).

**Figure 2:**
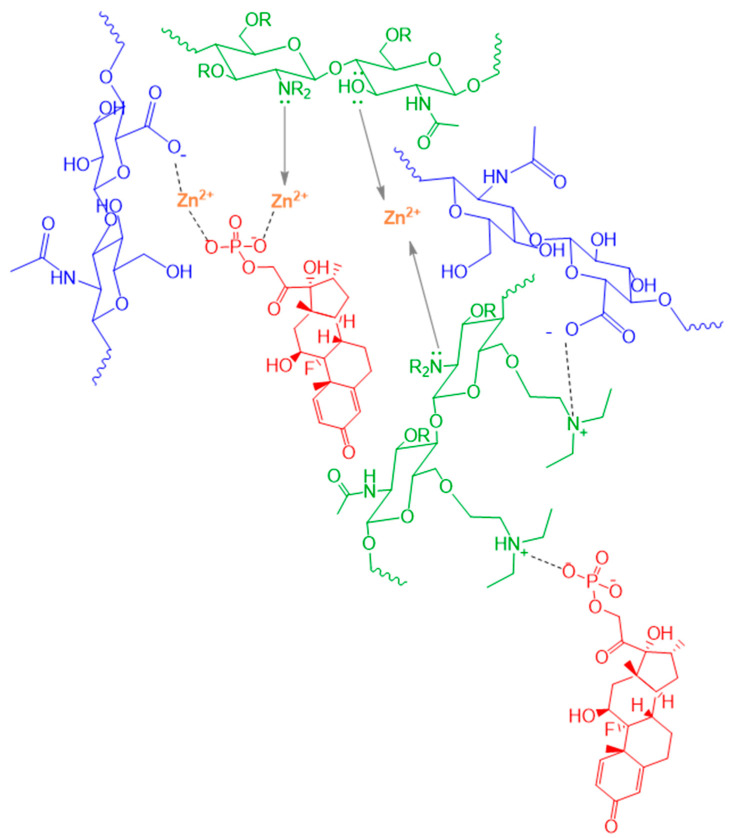
Some possible interactions in a system consisting of DexP, Zn^2+^, and polymers (DEAECS and HA).

In the second step, it was of interest to investigate the tri-component systems DexP-DEAECS-HA and DexP-HA-DEAECS by DLS. Furthermore, zinc sulfate solution was added to the tri-component systems (DexP-DEAECS-Zn-HA and DexP-HA-Zn-DEAECS) to better control the strength of the formed PECs, their size, and their surface charge. DEAECS is capable of chelating Zn^2+^ cations via amino and hydroxyl groups. The deprotonated amino groups are responsible for the complexing properties, while the protonated amino groups provide electrostatic interactions with both DexP anions and carboxylate groups of HA [32]. In addition, Zn^2+^ cations also bind to the phosphate group of DexP and the carboxylate group of HA [31]. Thus, particle formation is the result of complex interactions (Figure 2). The data obtained by the DLS method are shown in Table 1.

The order of polyelectrolyte mixing influenced both particle size and particle size distribution (PDI). The addition of zinc ions contributed to a decrease in particle size and an increase in particle size uniformity due to the cross-linking effect. Thus, the interaction of DexP with DEAECS resulted in the formation of large polymeric particles (DexP-DEAECS) with a size of 710 nm, a positive surface charge (ζ-potential of +17.9 mV), and a high PDI of 0.7. After the addition of HA (DexP-DEAECS-HA, Scheme 1a) to this system, the particle size was reduced to 518 nm, and PECs with a negative ζ-potential (−17.8 mV) were formed (Table 1). The introduction of zinc ions into the DexP-DEAECS system reduced both the particle size (to 604 nm) and the PDI to a value of 0.3 (DexP-DEAECS-Zn). The addition of HA to this system resulted in the formation of negatively charged (ζ-potential of −17 mV) PECs sized at 702 nm (DexP-DEAECS-Zn-HA, Scheme 1b).

By changing the mixing order of DEAECS and HA, we were able to obtain PECs with acceptable size (154 nm) and PDI (0.2), as well as a high ζ-potential of +26.8 mV (DexP-HA-DEAECS, Scheme 1c). The introduction of zinc ions into the mixture of DexP and HA resulted in the formation of large polydisperse particles (950 nm, PDI 0.6). Further treatment of the resulting system with DEAECS resulted in PECs with the desired size (244 nm) and narrow size distribution (PDI 0.1), as well as a suitable ζ-potential (24.4) to ensure colloidal stability of the system (DexP-HA-Zn-DEAECS, Scheme 1d). It should be noted that PDI values of 0.2 and below are generally considered acceptable for polymeric drug delivery systems [54]. The obtained particles retained their parameters, including size, ζ-potential, and PDI, for at least 24 h (Table 1).

The EE is an important parameter that determines the suitability of the process for PEC formation. As shown in Table 1, the order of mixing the components and the addition of zinc ions (cross-linking agent) affected the EE of DexP. DEAECS efficiently bound DexP (DexP-DEAECS, EE 77.8%), but when HA was added, some DexP was displaced, and the EE decreased to 37.8% (DexP-DEAECS-HA). In contrast, the addition of Zn^2+^ promoted an increase in EE to 86.7% due to additional binding of DexP molecules (DexP-DEAECS-Zn), which also led to an increase in EE to 58.5% when HA was added to the system (DexP-DEAECS-Zn-HA).

When DEAECS was added to the mixture of DexP and HA, the EE was only 10.5% (DexP-HA-DEAECS), apparently indicating a primary interaction between the polyelectrolytes. However, the introduction of the zinc ions increased the EE to 30.3% and 75.6% (DexP-HA-Zn and DexP-HA-Zn-DEAECS, respectively).

**Figure 3:**
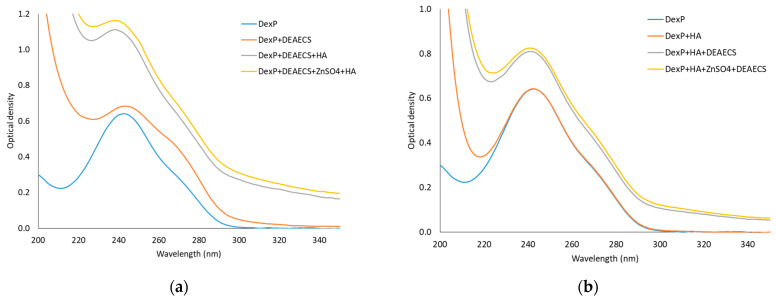
UV–VIS absorption spectra upon stepwise formation of DexP-DEAECS-Zn-HA (**a**) and DexP-HA-Zn-DEAECS (**b**).

UV–VIS spectra of three- and four-component systems show the interaction of DexP with DEAECS and a stepwise increase in the turbidity (baseline enhancement due to light scattering) of the PEC nanosuspension due to the formation of insoluble polymeric particles DexP-DEAECS-Zn-HA (Figure 3a) as well as DexP-HA-Zn-DEAECS (Figure 3b).

**Figure 4:**
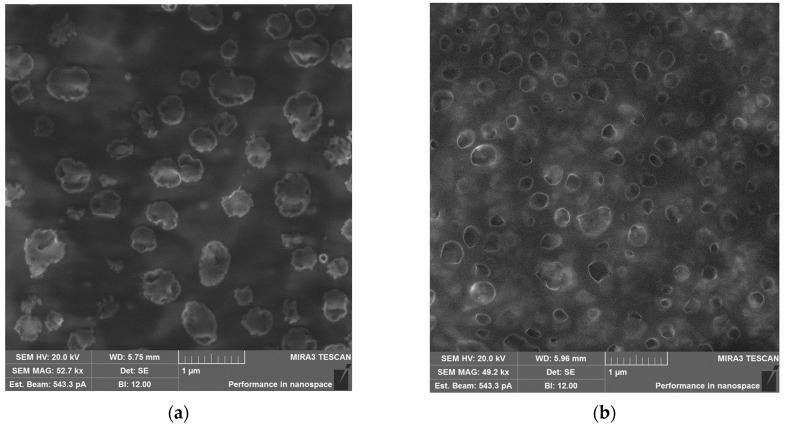
SEM images of DexP-DEAECS-Zn-HA (**a**) and DexP-HA-Zn-DEAECS (**b**).

SEM images of DexP-DEAECS-Zn-HA (Figure 4a) and DexP-HA-Zn-DEAECS (Figure 4b) showed the presence of spherical particles; the sizes of the PECs obtained in the solid state correspond to their hydrodynamic diameters, which is an indirect marker of the stiffness of zinc-containing PECs [48].

### 3.2. In Vitro DexP Release Kinetics from the PECs

**Figure 5:**
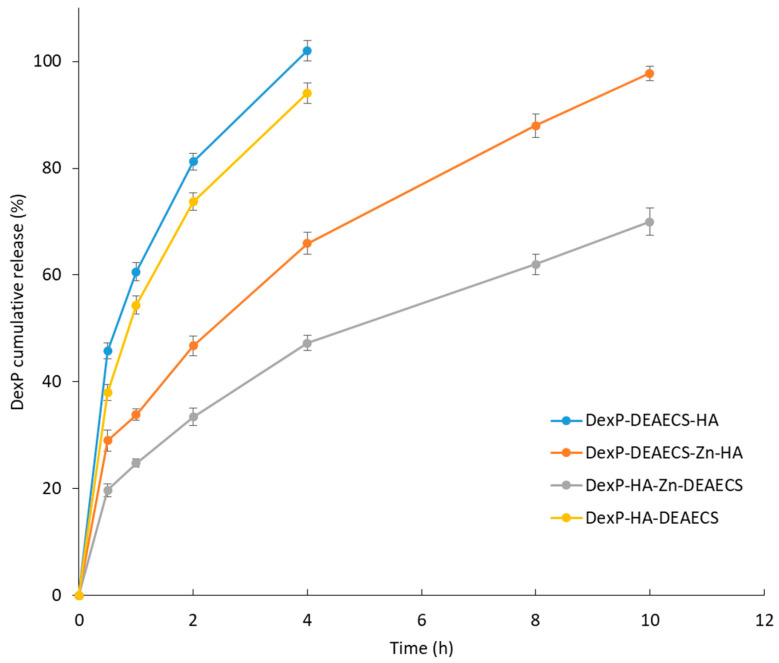
Release of DexP from the PECs in PBS at 32 °C. Data are presented as mean ± standard deviation (n = 3).

DexP in water interacts with polycationic DEAECS to form PECs as a result of the subsequent addition of polyanionic HA; the resulting PECs are further strengthened and stabilized by the introduction of zinc ions due to their cross-linking effect. However, under physiological conditions, due to the influence of pH and ionic strength, the bonds between the components are weakened, diffusion is increased, and drug molecules are released [30]. The in vitro kinetics of DexP release in PBS at 32 °C is shown in Figure 5.

DexP release from zinc-free PECs (DexP-DEAECS-HA and DexP-HA-DEAECS) was rapid within 2–4 h. In contrast, Zn^2+^-containing particles were characterized by delayed release, with a total of 98 and 70% DexP release in 10 h from DexP-DEAECS-Zn-HA and DexP-HA-Zn-DEAECS, respectively. The presence of DEAECS on the surface of polymeric particles prolonged drug release both by increasing the colloidal stability of PECs and by limiting diffusion due to ionic interactions of the polycationic polymer with the DexP anion. Thus, both the presence of zinc ions and an increase in the content of polycationic DEAECS in the system modified the release of DexP from the corresponding PECs. DexP-containing polymeric nanocarriers with these release profiles are attractive topical glucocorticoid delivery systems.

Assuming diffusion-controlled release, the cumulative DexP release curves were linearized according to the Higuchi and Korsmeyer–Peppas kinetic models [55,56]. The fitting parameters are shown in Table 2.

The kinetics of DexP release were in good agreement with both the Higuchi (4) and Korsmeyer–Peppas (5) models. The values of the release exponent (n ≤ 0.5) characterize the drug release mechanism as a Fickian diffusion (Case I transport) and diffusion-controlled process, which is typical for this type of polymeric particle [57].

### 3.3. Mucoadhesion of the PECs

**Figure 6:**
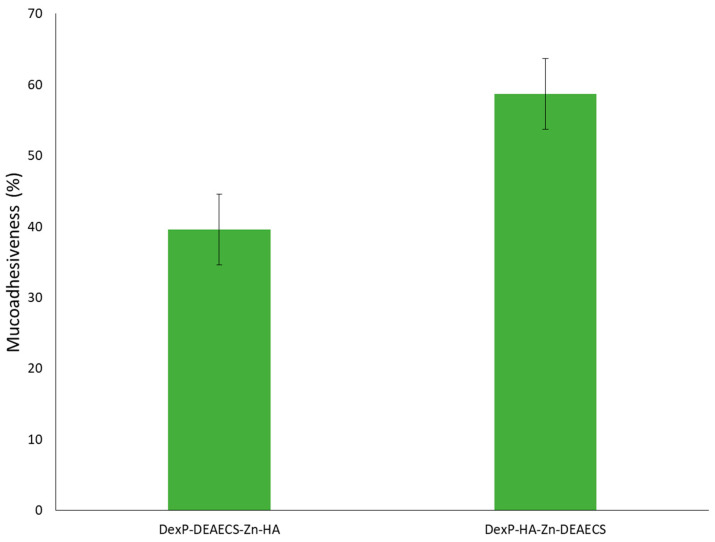
Mucoadhesiveness of DexP-DEAECS-Zn-HA and DexP-HA-Zn-DEAECS. Data are presented as mean ± standard deviation (n = 3).

Mucoadhesive ocular drug delivery systems adhere to the corneal mucosa, thereby increasing drug residence time and local bioavailability [58]. Both DEAECS and HA are capable of intermolecular interaction with various functional groups of mucin through hydrogen bonding and entanglement of polymer chains, as well as electrostatic bonding and hydrophobic interaction [58,59]. The mucoadhesion of DexP-DEAECS-Zn-HA and DexP-HA-Zn-DEAECS was tested as they were the most promising nanocarriers in terms of DexP release profile. The mucoadhesive properties were evaluated by the ability of the particles to bind mucin in aqueous solution. The amount of mucin adsorbed was measured by the change in free mucin concentration in the supernatant according to Equation (3). It was shown (Figure 6) that both DexP-DEAECS-Zn-HA and DexP-HA-Zn-DEAECS effectively bound mucin (mucoadhesive capacity was approximately 40 and 59%, respectively); however, the use of DEAECS-coated PECs with a positive surface charge increased the mucoadhesive capacity of the particles 1.5-fold. Thus, because of their ability to bind to mucin, zinc-containing PECs can prevent the rapid clearance of DexP from the corneal surface, indicating their promise in the treatment of inflammatory eye diseases.

### 3.4. Anti-Inflammatory Activity of the PECs

Our results showed that DexP, ZnSO_4_, HA-DEAECS, and DexP/Zn^2+^-containing PECs had no significant cytotoxic effect on TNFa-untreated THP-1 cells (Table 3). We also found that the combination treatment of THP-1 cells with TNFa and DexP, ZnSO_4_, HA-DEAECS, and DexP/Zn^2+^-containing PECs also had no significant effect on the viability of THP-1 cells. These results indicated that DexP, ZnSO_4_, HA-DEAECS, and DexP/Zn^2+^-containing PECs had no cytotoxic effects on THP-1 cells.

We then examined the effects of DexP, ZnSO_4_, HA-DEAECS, and DexP/ Zn^2+^-containing PECs on TNFa-induced cell surface CD54 expression by THP-1 cells (Table 4). The results confirmed that our TNFa stimulation effectively increased cell surface CD54 expression on THP-1 cells (4.61 (3.77; 5.55) MFI in negative controls vs. 0.77 (0.60; 0.86) MFI after 24 h in vitro co-culture with 2 ng/mL TNFa, *p* < 0.001). Interestingly, we found that two types of DexP-containing systems (DexP-HA-DEAECS and DexP-HA-Zn-DEAECS) increased CD54 expression on THP-1 cells without TNFa stimulation. Finally, we found that all DexP/ Zn^2+^-containing PECs significantly downregulated CD54 expression on TNFa-treated THP-1 cells, whereas ZnSO_4_ solution had no effect on CD54 expression. Taken together, our results indicate that DexP/Zn-containing PECs were effective in suppressing TNFa-induced THP-1 cell activation and exhibited anti-inflammatory activity in vitro.

## 4. Conclusions

We have developed a simple and convenient technique for obtaining DEAECS- and HA-based PECs with pronounced anti-inflammatory activity. The advantages of this technique are (i) easy preparation and mild preparation conditions, (ii) use of aqueous solutions, (iii) use of biocompatible and biodegradable polysaccharides, (iv) the possibility to control the size and surface charge of the formed PECs, (v) high EE, (vi) prolonged drug release within 10 h, and (vii) effective mucoadhesion.

It can be concluded that the key factor for the formation of stable particles of 200–300 nm size is the polyelectrolyte interaction between oppositely charged polymers upon the addition of DEAECS to HA. However, the obtained PECs have a low EE (10.5%) and a fast DexP release (within 2 h). The use of zinc ions as a cross-linking agent increased the EE to 75.6% and prolonged the drug release to 10 h.

The results indicate that the developed PECs are promising nanocarriers with desirable properties (including size, charge, EE, drug release profile, and mucoadhesion). Based on these data, we plan to extend our research to in vivo experiments with the goal of creating a topical DexP delivery system with enhanced bioavailability and improved therapeutic properties.

## Figures and Tables

**Table 1:** Formation conditions and properties of the PECs (mean ± standard deviation, n = 3).

Formulation and Mass Ratio of Initial Components	Scheme	Dh (nm)	PDI	ζ-Potential (mV)	EE (%)	DexP Content (μg/mg)	Zn^2+^ Content (%)
DexP-DEAECS 1:5	1a	710 ± 318	0.7	+17.9 ± 0.7	77.8	-	-
DexP-DEAECS-HA 1:5:10	1a	518 ± 124 514 ± 136 *	0.5 0.5 *	−17.8 ± 0.2 −18.1 ± 0.3 *	37.8	24.6	-
DexP-DEAECS-Zn 1:5:1	1b	604 ± 162	0.3	+18.1 ± 0.5	86.7	-	-
DexP-DEAECS-Zn-HA 1:5:1:10	1b	702 ± 158 714 ± 142 *	0.3 0.3 *	−17.0 ± 0.2 −17.2 ± 0.4 *	58.5	35.7	2.2 ± 0.7
DexP-HA-DEAECS 1:5:10	1c	154 ± 28 155 ± 34 *	0.2 0.2 *	+26.8 ± 0.5 +26.1 ± 0.7 *	10.5	7.0	-
DexP-HA-Zn 1:5:1	1d	950 ± 253	0.6	−21.4 ± 0.6	30.3	-	-
DexP-HA-Zn-DEAECS 1:5:1:10	1d	244 ± 56 256 ± 61 *	0.1 0.1 *	+24.4 ± 0.3 +25.3 ± 0.2 *	75.6	45.4	2.3 ± 0.8

* Particle parameters after storage for 24 h as an aqueous dispersion at room temperature.

**Table 2:** Fitting parameters of the kinetic models of DexP release.

Formulation	Kinetic Model *				
	Higuchi Q = K_H_t^0.5^ (4)		Korsmeyer-Peppas Q = K_KP_t^n^ (5)		
	K_H_	R^2^	K_KP_	n	R^2^
DexP-DEAECS-HA	54.9	0.9743	65.0	0.50	0.9986
DexP-DEAECS-Zn-HA	31.9	0.9906	37.1	0.40	0.9966
DexP-HA-DEAECS	49.8	0.9865	51.0	0.50	0.9943
DexP-HA-Zn-DEAECS	22.6	0.9915	25.5	0.43	0.9993

* Q is the cumulative DexP release (%); K_H_ is the Higuchi constant; K_KP_ is the release rate constant; n is the release exponent, t is the time.

**Table 3:** Cell viability (the corresponding numbers of live YO-PRO-1negPIneg cells) staining of THP-1 cells treated with DexP, ZnSO_4_, HA-DEAECS, and DexP/ Zn^2+^-containing PECs for 24 h as detected by YO-PRO-1/PI staining (n = 9). Data are presented as median and interquartile range, Me (Q25; Q75).

Sample	no TNFa Added	2 ng/mL TNFa
Negative control	95.94 (95.63; 96.30)	96.08 (95.65; 96.28)
DexP-HA-DEAECS	96.44 (96.10; 96.63)	96.20 (95.70; 96.31)
DexP-DEAECS-Zn-HA	96.32 (96.21; 96.63)	96.17 (95.93; 96.34)
HA-DEAECS	96.07 (95.61; 96.34)	96.06 (95.84; 96.25)
ZnSO_4_	96.60 (96.23; 96.75)	96.09 (95.83; 96.40)
DexP-HA-Zn-DEAECS	96.25 (96.03; 96.49)	96.09 (95.95; 96.27)
DexP	96.46 (96.18; 96.48)	95.74 (95.50; 96.13)
DexP-DEAECS-HA	96.20 (96.06; 96.39)	96.22 (96.01; 96.35)

**Table 4:** CD54 expression by THP-1 cells in vitro treated with DexP, ZnSO_4_, HA-DEAECS, and DexP/ Zn^2+^-containing PECs. Data are presented as median and interquartile range, Me (Q25; Q75) (n = 6); data are presented as CD54 MFI.

Sample	no TNFa Added	2 ng/mL TNFa
Negative control	0,77 (0,60; 0,86)	4,61 (3,77; 5,55)
DexP-HA-DEAECS	1.01 (0.81; 1.03) **	2.24 (2.15; 2.41) **
DexP-DEAECS-Zn-HA	0.80 (0.65; 1.17)	1.90 (1.84; 2.37) **
HA-DEAECS	0.66 (0.61; 0.68)	2.63 (2.47; 3.63) *
ZnSO_4_	0.64 (0.61; 0.64)	3.84 (3.40; 4.06)
DexP-HA-Zn-DEAECS	0.95 (0.89; 1.36) *	2.71 (2.44; 3.05) **
DexP	0.62 (0.59; 0.67)	1.26 (1.09; 1.63) **
DexP-DEAECS-HA	0.80 (0.74; 0.87)	1.93 (1.78; 2.00) **

* and **—the differences from the negative control (THP-1 cells without addition of DexP, ZnSO_4_, and DexP/ Zn^2+^-containing PECs) were significant at *p* < 0.05 and *p* < 0.01, respectively, according to the non-parametric Mann–Whitney U test.

## Data Availability

The data are contained within the article.
